# *Mycobacterium tuberculosis* Glyceraldehyde-3-Phosphate Dehydrogenase (GAPDH) Functions as a Receptor for Human Lactoferrin

**DOI:** 10.3389/fcimb.2017.00245

**Published:** 2017-06-08

**Authors:** Himanshu Malhotra, Anil Patidar, Vishant M. Boradia, Rajender Kumar, Rakesh D. Nimbalkar, Ajay Kumar, Zahid Gani, Rajbeer Kaur, Prabha Garg, Manoj Raje, Chaaya I. Raje

**Affiliations:** ^1^Cell Biology and Immunology, Council of Scientific and Industrial Research-Institute of Microbial TechnologyChandigarh, India; ^2^Department of Biotechnology, National Institute of Pharmaceutical Education and ResearchPunjab, India; ^3^Department of Pharmacoinformatics, National Institute of Pharmaceutical Education and ResearchPunjab, India

**Keywords:** *Mycobacterium tuberculosis*, iron, transferrin, lactoferrin, glyceraldehyde-3-phosphate dehydrogenase (GAPDH)

## Abstract

Iron is crucial for the survival of living cells, particularly the human pathogen *Mycobacterium tuberculosis (M.tb)* which uses multiple strategies to acquire and store iron. *M.tb* synthesizes high affinity iron chelators (siderophores), these extract iron from host iron carrier proteins such as transferrin (Tf) and lactoferrin (Lf). Recent studies have revealed that *M.tb* may also relocate several housekeeping proteins to the cell surface for capture and internalization of host iron carrier protein transferrin. One of the identified receptors is the glycolytic enzyme Glyceraldehyde-3-phosphate dehydrogenase (GAPDH). This conserved multifunctional protein has been identified as a virulence factor in several other bacterial species. Considering the close structural and functional homology between the two major human iron carrier proteins (Tf and Lf) and the fact that Lf is abundantly present in lung fluid (unlike Tf which is present in plasma), we evaluated whether GAPDH also functions as a dual receptor for Lf. The current study demonstrates that human Lf is sequestered at the bacterial surface by GAPDH. The affinity of Lf-GAPDH (31.7 ± 1.68 nM) is higher as compared to Tf-GAPDH (160 ± 24 nM). Two GAPDH mutants were analyzed for their enzymatic activity and interaction with Lf. Lastly, the present computational studies offer the first significant insights for the 3D structure of monomers and assembled tetramer with the associated co-factor NAD^+^. Sequence analysis and structural modeling identified the surface exposed, evolutionarily conserved and functional residues and predicted the effect of mutagenesis on GAPDH.

## Introduction

Iron acquisition is vital for the survival of *Mycobacterium tuberculosis*, the causative agent of tuberculosis. In order to survive and replicate within the iron restricted intracellular environment of the host cell, these pathogens utilize multiple strategies to pilfer iron from host resources. In the mammalian host iron remains tightly sequestered to either storage proteins or to transport proteins such as Tf and Lf. One strategy used by *M.tb* to acquire iron is to synthesize high affinity chelators known as siderophores (De Voss et al., 1999; Ratledge, 2004; Banerjee et al., 2011). Recently an alternate siderophore–independent pathway has been identified wherein human holo-Tf is directly captured at the bacterial surface and subsequently internalized. The *M.tb* Tf receptors identified included several conserved proteins namely Glyceraldehyde-3-phosphate dehydrogenase (GAPDH, Rv1436); Lactate dehydrogenase (Rv1872c); Iron regulated Elongation factor tu (Rv0685); Acyl desaturase (Rv0824c); 50S ribosomal protein L2rplB (Rv0704); 50S ribosomal protein L1rplA (Rv0641) (Boradia et al., 2014). The essential house-keeping glycolytic enzyme GAPDH operates as a multifunctional protein in both eukaryotes (Sirover, 1999) and prokaryotes including *M.tb* (Bermudez et al., 1996; Carroll et al., 2010). Earlier GAPDH has been identified as a dual receptor for both Tf and Lf in mammalian cells (Raje et al., 2007; Rawat et al., 2012). GAPDH therefore plays analogous roles in Tf acquisition of iron by *M.tb* as well as its human host (Boradia et al., 2014).

Tf is predominantly present in the blood and is involved in iron transport to cells, while Lf is abundantly present in human milk, mucosal secretions, and neutrophil secretory granules. The two iron carrier proteins Lf and Tf share significant sequence identity (60%) and have a conserved bi-lobed structure that each bind single atom of Fe^3+^. Lf plays a vital role in iron sequestration, transport, and as an immunomodulator. Several studies have revealed that it plays a protective role by enhancing phagocytosis, inhibiting biofilm formation, and preventing of microbe-host interactions (Testa, 2002; Siqueiros-Cendón et al., 2014).

While Tf iron acquisition in *M.tb* has been extensively assessed, limited information is available regarding acquisition of Lf iron in this pathogen. A few previous reports have indicated that *M.tb* bacilli in culture and intracellular bacilli can acquire Lf associated iron. Infact these studies have shown that iron is acquired several fold more efficiently from Lf than Tf (Olakanmi et al., 2004), however the exact mechanisms involved remain unknown.

In the current study using *in vitro* and cell based models, we demonstrate for the first time that *M.tb* acquires iron by utilizing GAPDH as receptor for Lf. Ligand binding analysis demonstrated that GAPDH had a greater affinity for Lf than Tf. Lf uptake by *M.tb* was evident in virulent, attenuated, and even siderophore negative *M.tb* strains with Lf being trafficked to the intraphagosomal bacilli. Taken together, these findings suggest that iron acquisition from Lf is independent of the siderophore pathway. Mutagenesis and *in silico* studies provided an insight into the structure and function of GAPDH. This study also provides the first 3D model for *M.tb* GAPDH and confirms that enzyme activity and Lf binding are unrelated.

## Materials and methods

### Plasmids and strains

The *M.tb* H37Rv ΔmbtB::hyg, siderophore knockout strain was received as a kind gift from Dr. CE Barry, Tuberculosis Research Section, Laboratory of Host Defenses, National Institute of Allergy and Infectious Disease, Rockville, Maryland (De Voss et al., 2000). *M.tb* H37Ra and H37Rv strains were transformed with pSC301 plasmid (Cowley and Av-Gay, 2001) to generate the *M.tb* H37Ra-GFP and *M.tb* H37Rv-GFP strains. *M.tb* H37Ra strains expressing mCherry or GAPDH-mCherry were prepared previously in our laboratory (Boradia et al., 2014).

The full length *M.tb* GAPDH gene was cloned and expressed in *M.tb* H37Ra to obtain recombinant wild type GAPDH (wt rGAPDH) as described previously (Boradia et al., 2016). During plasmid screening two point mutations were detected (i) an Arginine to Serine mutation at position 142 (N142S) and (ii) a Proline to Leucine mutation at position 295 (P295L). Each plasmid was individually transformed into *M.tb* H37Ra using standard methods (Wards and Collins, 1996), recombinant proteins were purified as described previously (Boradia et al., 2016), the mutant proteins are referred to as rGAPDH(N142S) and rGAPDH(P295L) respectively.

### Conjugation of proteins

Human Lf (Sigma Chemical Co.) was biotinylated using Sulfo-NHS-LC-Biotin as per the manufacturer's instructions (Pierce). Hilyte Fluor (HF)-488 was conjugated to Lf using AnaTag™ HiLyte Fluor™ 488 Protein Labeling Kit according to the manufacturer's instructions.

### Surface binding of Holo-Lf-HiLyte fluor488 (Lf-HF-488) by FACS analysis

*M.tb* H37Ra cells were cultured to log phase, 2 × 10^8^ cells were used per assay. Cells were washed with PBS and blocked for 1 h at 4°C with PBS containing 2% BSA. Cells were then incubated with 10 μg Lf-HF488 alone or in the presence of 100-fold excess unlabeled human holo-Lf at 4°C for 2 h. Finally, cells were washed extensively with PBS and the fluorescence data of 10^4^ cells per assay was acquired using a Guava 8HT flow cytometer. Experiments were repeated thrice, statistical significance was determined by Student's *t*-test.

### Cell surface binding of Lf-Gold by transmission electron microscopy (TEM)

To assess Lf binding, *M.tb* H37Ra bacilli were cultured to log phase, 2 × 10^8^ cells were used per assay. Cells were washed with PBS and blocked in PBS containing 2% casein at 4°C for 1 h. Cells were then incubated with 10 μg of Lf- gold (20 nm) in PBS containing 0.2% casein at 4°C for 2 h. Cells were washed and fixed in Karnovsky's fixative for 30 min, followed by two washes with 5 mM NaCl. Finally, cells were resuspended in PBS, placed on carbon coated grids and viewed in a JEOL 2100 Transmission Electron Microscope (TEM). As a negative control, samples were similarly incubated with streptavidin gold conjugate (20 nm, Sigma) instead of Lf. Gold particles were prepared as described previously (Boradia et al., 2014).

### Lf surface binding and uptake into *M.tb* H37Ra bacilli overexpressing GAPDH mCherry and mCherry

*M.tb* H37Ra mCherry and *M.tb* H37Ra GAPDH mCherry expressing cells were cultured to log phase, 1 × 10^8^ cells were used per assay. Cells were washed with iron free Sauton's media and blocked for 1 h at 4°C with 2% BSA in PBS. Cells were then incubated with Lf conjugated to Alexa fluor 647 (Lf-A647, 10 μg per 100 μl of iron free Sauton's media) at 4°C for 2 h. Finally, cells were washed extensively with iron free Sauton's media and fluorescence data of 10^4^ cells per assay was acquired using a BD FACS Verse instrument. Experiments were repeated twice in duplicates, statistical significance was analyzed by Student's *t*-test. Data is represented as an overlay histogram and as a bar graph of MFI values.

For uptake experiments, cells were processed as before, blocking was done with SFM containing 2% BSA for 30 min at 4°C. Subsequently, bacteria were incubated with 10 μg Lf-A647 at 37°C for 2 h. Cells were then washed twice and treated with 0.1% pronase at 4°C for 5 min on ice to remove any residual surface bound proteins. Finally, cells were washed extensively with iron free Sauton's media and fluorescence data of 10^4^ cells per assay was acquired using a BD FACS Verse instrument. Experiments were repeated twice in duplicates, statistical significance was analyzed by Student's *t*-test. Data is represented as an overlay histogram and as a bar graph of MFI values.

### Evaluation of Lf-Iron uptake

Log phase *M.tb* H37Ra mCh and *M.tb* H37Ra GAPDH-mCh cells, (2 × 10^8^ cells / assay) were washed twice with Sauton's media without iron followed by incubation with the same media for 2 h at 37°C. Cells were then washed and incubated for a further 1 h at 37°C with media containing 50 μg of Lf-^55^Fe in 100 μl Sauton's media without iron. Cells were washed thrice with iron-free media, centrifuged, and resuspended in 3.0 ml of scintillation fluid. The radioactivity associated with the cells was measured using a beta counter (Perkin Elmer), counts were normalized to cell number. Statistical significance was estimated by Student's *t*-test (*n* = 6) (Boradia et al., 2014).

### FRET analysis of Lf-GAPDH interaction

Log phase *M.tb* H37Ra mCh and *M.tb* H37Ra GAPDH-mCh cells, (2 × 10^8^ cells/assay) were washed and blocked with 2% BSA followed by incubation with 20 μg Lf-FITC in 100 μl PBS, for 2 h at 4°C. Samples were fixed in 4% buffered paraformaldehyde for 20 min and FRET analysis was carried out by the acceptor photobleaching method on a Nikon A1R confocal microscope as described previously (Raje et al., 2007). The intensities of a total of 70 regions of interest from multiple, randomly selected fields were measured before and after photobleaching, and FRET efficiency was calculated (Boradia et al., 2014). Statistical analysis was done by Student's *t*-test.

### Lf binding on *M.tb* H37Rv and *M.tb* H37RvΔmbtB::hyg bacilli

*M.tb* H37Rv and *M.tb* H37Rv ΔmbtB::hyg strains were cultured to log phase, and 1 × 10^8^ cells were used per assay. Cells were washed with phosphate-buffered saline (PBS) and blocked for 1 h at 4°C with 2% BSA in SFM (serum free media). Cells were then incubated with Lf-A647 (10 μg per 100 μl of SFM) alone or in the presence of 200-fold excess unlabeled human Lf at 4°C for 2 h. Finally, cells were washed extensively with SFM and fluorescence data of 10^4^ cells per assay was acquired using a BD FACS Accuri instrument. Experiments were repeated thrice in duplicates. Statistical significance was analyzed by Student's *t*-test.

### Lf uptake in *M.tb* H37Rv and *M.tb* H37RvΔmbtB::hyg bacilli

Cells (1 × 10^8^ bacilli/assay) were blocked with SFM containing 2% BSA for 30 min at 4°C. Subsequently, bacteria were incubated with 10 μg Lf-A647. After incubation at 37°C for 2 h, cells were treated with 0.1% pronase at 4°C for 5 min to remove any residual surface bound proteins. Cells were washed extensively with SFM and fluorescence data of 10^4^ cells per assay was acquired using a BD FACS Verse instrument, experiments were repeated thrice in triplicates. Statistical significance was analyzed by Student's *t*-test.

### Trafficking of Lf to intracellular bacilli

The human macrophage cell line, THP-1, was utilized as an infection model. To study trafficking of Lf to bacteria resident within macrophages, THP-1 cells were plated into Matek glass bottom petri dishes (3 × 10^5^ cells per dish). Cells were activated with 25 ng/ml PMA (Sigma) for 24 h and rested for an additional 24 h. Cells were then shifted to antibiotic-free RPMI media containing 10% fetal calf serum and infected with bacilli at a ratio of 1:20 (THP-1: bacteria) using log phase cultures of *M.tb* H37Rv-GFP, *M.tb* H37Ra mCherry, or *M.tb* H37Ra GAPDH-mCherry. After 6 h, cells were washed with serum-free media (SFM) to remove non-phagocytosed bacteria. A second wash with SFM was carried out after 24 h of infection and cells were shifted to SFM for 30 min at 37°C. Cells were then washed and incubated with 10 μg of Lf-A647 in 100 μl SFM media at 4°C for 30 min. Subsequently, for internalization of bound Lf cells were shifted to 37°C, 5% CO_2_ for 1 h. After extensive rinsing with SFM, cells were fixed with 2% paraformaldehyde for 15 min and imaged on a Nikon A1R confocal microscope using a 60X oil immersion objective and an aperture of 1 airy unit.

### Live cell imaging of *M.tb* H37Ra-GFP infected THP-1 cells

THP-1 cells were plated and processed for infection with log phase cultures of *M.tb* H37Ra-GFP and incubation with Lf-A647 as described above. Subsequently cells were imaged live on a Nikon A1R confocal microscope equipped with an Okolab bold line water-jacket cryo topstage CO_2_ incubator system (Okolab Italy).

### Immuno-gold labeling TEM to detect internalized Lf in *M.tb* H37Rv

Cells (2 × 10^9^ bacilli per sample) were blocked with SFM containing 2% BSA for 30 min at 4°C. Subsequently, bacteria were incubated with 1 mg of Lf-conjugated with 20 nm gold particles for 2 h at 37°C. Subsequently cells were treated with 0.1% pronase at 4°C for 10 min to remove any residual surface bound proteins. Cells were then fixed, embedded in epoxy resin and ultrathin sections cut for visualization in TEM essentially as described previously (Boradia et al., 2014).

### Expression and purification of mutants and GAPDH enzymatic activity

Both mutant forms of the protein were expressed and purified as described previously for wt rGAPDH (Boradia et al., 2016). Purification was confirmed by western blotting, detection was done after incubation with Mouse anti-His (Sigma) (1:3,000) for 1 h followed by incubation with anti-Mouse IgG HRP (Sigma) (1:8,000) for 1 h. The enzyme activity of wt rGAPDH, rGAPDH(N142S), and rGAPDH(P295L) purified from cytosol fraction was studied by measuring the increase in the absorbance at 340 nm due to oxidative reduction of NAD^+^ to NADH. The reaction mixture containing 200 μl of enzyme assay buffer (50 mM HEPES, 10 mM sodium arsenate, and 5 mM EDTA, pH 8.5), 1 mM NAD^+^ and 2 mM glyceraldehyde-3-phosphate (G-3-P) was added to wells containing 100 ng of each purified enzyme at 25°C. Enzyme activity was measured at 340 nm for 5 min on a Tecan Infinity M200 multimode microplate reader. Negative controls were set up without the specific substrate G-3-P and their values were subtracted from the final absorbance. The experiment was repeated thrice in triplicates. The enzymatic activity of wt rGAPDH was taken as 100% and data was plotted as % residual activity ± *SD*. Statistical analysis was done using Student's *t*-test.

### Interaction of GAPDH mutants with human Lf

Lf (3 μg), BSA (5 μg), wt rGAPDH (1 μg), rGAPDH (N142S) (1 μg), or rGAPDH (P295L) (1 μg) were resolved on 10% SDS-PAGE and then transferred to nitrocellulose membrane. The membrane was then blocked with 5% BSA and probed with 10 μg/ml of either wt rGAPDH, rGAPDH(N142S) or rGAPDH(P295L) for 2 h, followed by incubation with mouse anti-His (Sigma) (1:3,000) for 1 h and anti-mouse IgG HRP (Sigma) (1:8,000) for 1 h. Finally, blots were washed and developed with TMB/H_2_O_2_; the experiment was repeated three times.

### Analysis of Lf-GAPDH interaction by far western blotting

Recombinant *M.tb* H37Rv GAPDH, wild type (wt rGAPDH) was prepared as described previously (Boradia et al., 2016). Lf, BSA (3 μg each) and wt rGAPDH (400 ng) were run on a 10% SDS-PAGE and transferred to nitrocellulose membrane. The membrane was probed with 10 μg/ml of wt rGAPDH for 2 h, followed by incubation with rabbit anti-GAPDH (1:1,000) for 1 h and anti-rabbit IgG HRP (1:16,000) for 1 h; blots were developed with TMB/H_2_O_2_, experiments were repeated thrice.

### Determination of affinity of interaction by solid phase binding assay

The affinity of the GAPDH and Lf interaction was estimated using a plate based solid phase binding assay. Polystyrene wells were coated overnight at 4°C using 2 μg/well of either wt rGAPDH, rGAPDH(N142S) or rGAPDH(P295L) mutant proteins in PBS (pH 7.4) and blocked using 5% cold fish skin gelatin prepared in PBS for 4 h at room temperature. GAPDH coated wells were then incubated at 25°C for 2 h with increasing concentrations (7.5−1,000 nM) of biotinylated Lf in PBS containing 1% fish skin gelatin. Control wells were incubated with buffer alone. Extensive washing with PBST was done out to remove any unbound Lf. Bound Lf was detected using primary antibody Mouse anti-Biotin (1:5,000), secondary antibody anti-Mouse IgG HRP (1:20,000) followed by the addition of TMB-H_2_O_2_ for development of color. Data was plotted as absorbance at 650 nm vs. concentration of biotinylated Lf after subtracting OD values of control wells. Experiments were performed thrice (with triplicates) and data was plotted for non-linear fit, one-site specific binding using Graph Pad Prism®.

### Sequence conservation analysis of GAPDH

Initial evolutionary conservation sequence analysis to identify functionally and structurally important residues in *M.tb* GAPDH were performed by the ConSurf Program (Ashkenazy et al., 2010, 2016). The primary sequence of wild type, N142S and P295L mutants as well as *M.tb* GAPDH modeled structure were considered. For query sequences and structure the CSI-BLAST (Context-Specific Iterated-Basic Local Alignment Search Tool) homolog search algorithm were used with 0.00001 as E-value cutoff, set at a maximal 95% identity and minimal identity of 35% between sequences for homology searches. The protein database UNIREF-90 (http://www.uniprot.org/help/uniref) was utilized; all other parameters were kept at default values for calculation of conservation scores. The primary goal was to reveal the highly conserved regions and functionally and structurally important residues in *M.tb* GAPDH.

### Comparative modeling of GAPDH

GAPDH is a homo-tetramer composed of four identical 36 kDa subunits. The structure of GAPDH alongwith its associated cofactor NAD^+^ has been reported for other homologous organisms. The sequence of *M.tb* H37Rv GAPDH was retrieved from NCBI (GenBank: CAB09248.1), the protein sequence is composed of 339 amino acids. The sequence was analyzed by NCBI protein BLAST search against Protein Data Bank (PDB) in order to get a template with a suitable identity. Tetrameric GAPDH from *Bacillus stearothermophilus* (PDB ID: 1GD1) was identified as the best template and was utilized for homology modeling of GAPDH using Modeller9v program (Webb and Sali, 2014).

### Assembly of monomer units and analysis of mutants

GAPDH homo-tetramer assembled from four identical 36 kDa subunits was done using superimposition tool of Chimera program (Pettersen et al., 2004; Meng et al., 2006). The template (PDB ID: 1GD1) was assembled into its homo-tetrameric structure, each unit of modeled *M.tb* GAPDH was then superimposed upon this template. The assembled model was energy minimized to correct the unfavorable bond lengths, bond angles, torsion angles, and contacts. Subsequently, the final model was verified and validated using RAMPAGE Ramachandran plot analysis server (http://mordred.bioc.cam.ac.uk/~rapper/rampage.php) and native protein folding energy assessment by PRoSA program (https://prosa.services.came.sbg.ac.at/prosa.php). The position of the two mutations was analyzed in context of the GAPDH tetramer and its proximity to the NAD^+^ binding site.

### Analysis of mutants by computational methods

Two selected mutations (N142S and P295L) were experimentally tested for the two functions attributed to GAPDH i.e., (i) enzyme activity and (ii) ability to bind Lf. Of these Asparagine 142 is a conserved residue and mutation to serine resulted in a loss of enzyme activity. However, both mutants retained their ability to bind Lf in far-western blots. Both mutants were explored to predict the effect of these alterations on protein stability and proximity to substrate binding site. The effect of mutations on protein stability were analyzed using DUET (Pires et al., 2014), mCSM and Site Directed Mutator (SDM) (Worth et al., 2011).

## Results

### Cell surface binding of Lf on *M.tb* H37Ra cells

**Figure 1:**
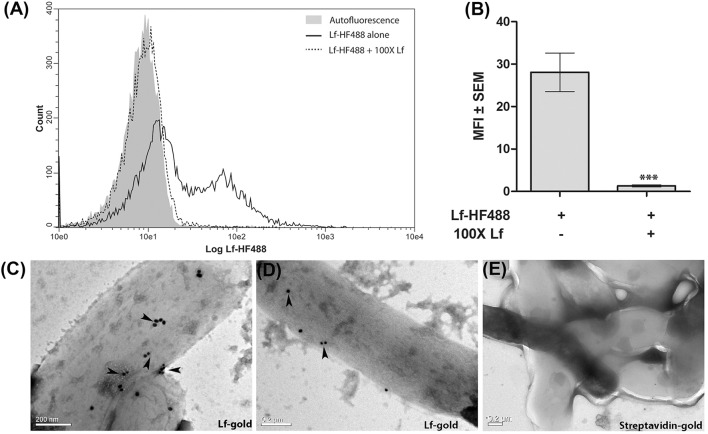
Surface binding of Lf. **(A,B)** FACS analysis indicates specific binding of Lf-HF488 on the surface of *M.tb* H37Ra cells **(A)** Overlay histogram plot of one representative experiment indicating log fluorescence of Lf-HF488. **(B)** Bar graph depicting MFI ± SEM. **(C–D)** TEM images reveal the binding of Lf conjugated gold particles (indicated by arrow heads) and **(E)** Control cells treated with control Streptavidin gold particles, demonstrate no surface binding of gold particles. Scale bar 0.2 μM. Multiple fields were analyzed with each sample, representative images are shown.

Flow cytometry clearly demonstrated the binding of Lf to *M.tb* H37Ra cells which was significantly inhibited (*p* < 0.0001) in the presence of 100-fold excess of unlabeled Lf, indicative of a specific interaction (Figures 1A,B). The binding of Lf-gold particles conjugated particles to *M.tb* H37Ra was confirmed by TEM analysis (Figures 1C–E).

### GAPDH mediated Lf-Iron delivery

**Figure 2:**
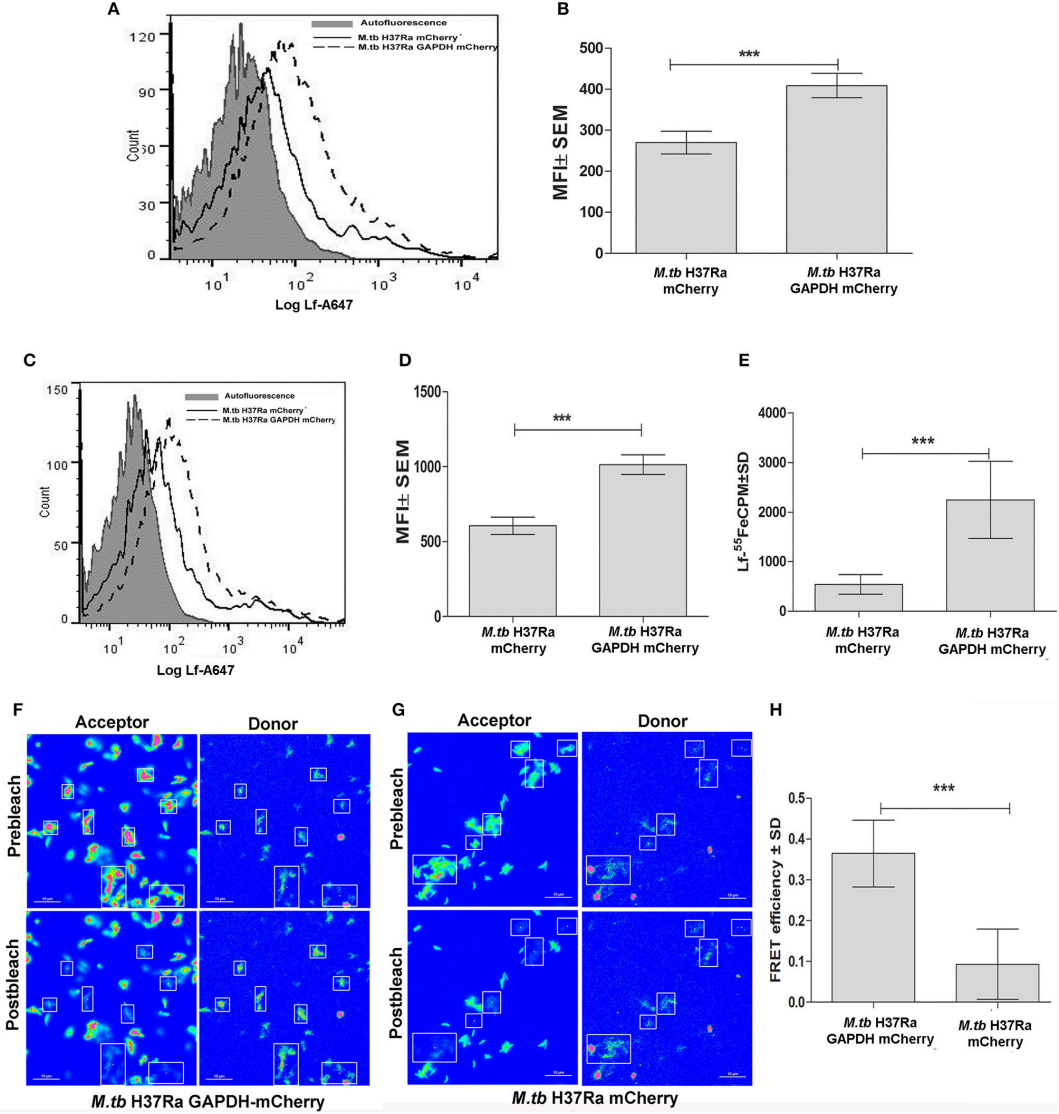
Enhanced GAPDH promotes binding, uptake and iron acquisition via Lf: FACS analysis indicates **(A–B)** binding of Lf-A647 **(C–D)** uptake of labeled Lf **(E)** Iron uptake in *M.tb* H37Ra GAPDH mCherry and mCherry strains. (*p* < 0.0005; *n* = *6*) **(F)** Acceptor photobleaching FRET analysis of the GAPDH-Lf interaction on the surface of *M.tb* H37Ra cells. Upper panels indicate the pre-bleach images of acceptor and donor respectively, while lower panels indicate post-bleach images of acceptor and donor. An increase in donor (Lf-FITC) signal is observed on photobleaching of acceptor (GAPDH-mCh) **(G)** A control experiment using *M.tb* H37Ra cells expressing mCh alone. **(H)** Quantitative analysis of FRET signal of 70 regions of interest from multiple, randomly selected fields were analyzed from both test and control samples. The FRET efficiency change ± s.d. of the donor and acceptor signals after photobleaching GAPDH-mCh and mCh was determined using NIS-Elements (Nikon) software. The % intensity change of the Lf-FITC signal was significantly higher in cells expressing GAPDH-mCh as compared to mCh alone (^***^p < 0.0001, Student's *t*-test, *n* = 70).

*M.tb* H37Ra strains overexpressing GAPDH-mCherry (GAPDH-mCh) or mCherry (mCh) alone were utilized to confirm whether Lf iron is GAPDH dependent. The overexpression of GAPDH in *M.tb* H37Ra resulted in enhanced binding (Figures 2A,B) and uptake of Lf when compared to control cells that over express mCherry alone (*p* < 0.0005) (Figures 2C,D). A correlated increase in iron uptake was also evident in the *M.tb* GAPDH-mCh strain as compared to the mCh strain (Figure 2E).

### Confirmation of GAPDH-Lf interaction on the *M.tb* cell surface

To confirm that GAPDH and Lf interact on the bacterial cell surface we carried out acceptor photobleaching Förster resonance energy transfer (FRET) analysis using *M.tb* H37Ra cells expressing GAPDH-mCh or mCh alone. This assay is based on the principle of two interacting partners which are fluorescently tagged. The fluorophores are selected such that the emission spectra of the donor overlaps with the absorption spectra of the acceptor. In the current study if Lf-FITC and GAPDH-mCherry interact, when excitation is done with the wavelength specific for Lf-FITC a strong fluorescence of mCherry is evident. When mCherry is destroyed (by photobleaching) an increase in donor signal is visible because the acceptor is no longer available to parasitize the light emitted from Lf-FITC (Kenworthy, 2001). In the present study a significant increase in donor (Lf-FITC) fluorescence intensity was observed upon photobleaching of acceptor (mCh) in the *M.tb* H37Ra GAPDH-mCh strain (Figures 2F,H) as compared to the strain expressing mCh alone (Figures 2G,H). The occurrence of FRET between the GAPDH and Lf indicates that they are at proximity of 1–10 nm (Kenworthy, 2001).

### Cell surface binding and uptake of Lf by *M.tb* H37Rv and *M.tb* H37Rv ΔmbtB::hygr strain

**Figure 3:**
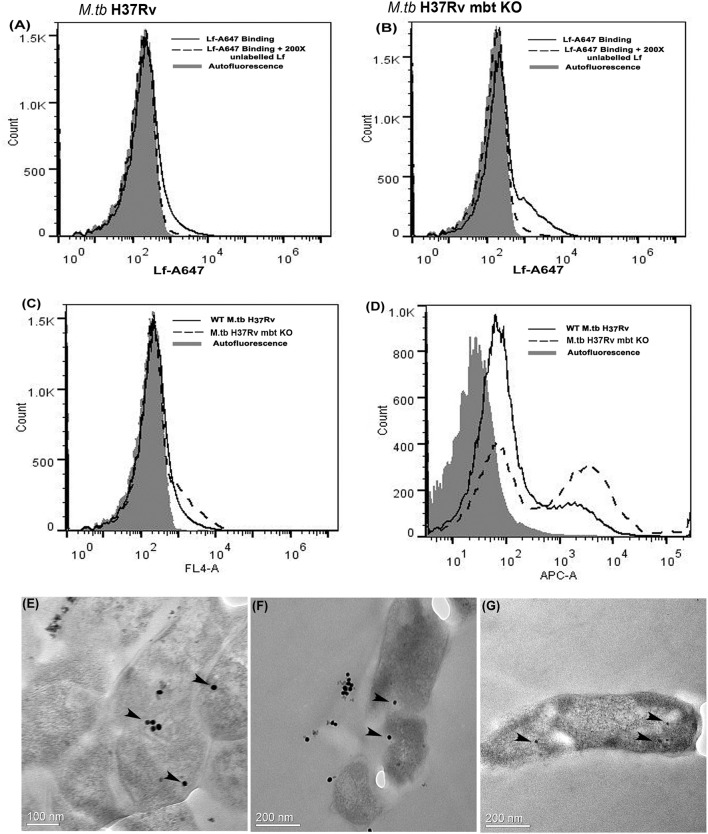
Lf surface binding and internalization in *M.tb* H37Rv strains: FACS analysis indicates specific binding of Lf-A647 on the surface of **(A)** *M.tb* H37Rv cells and **(B)** *M.tb* H37Rv mbt KO strain. **(C)** Comparative binding of Lf-A47 on the surface of both strains **(D)** Internalization of Lf-A647 in *M.tb* H37Rv and H37Rv mbt KO strain. **(E–G)** TEM images reveal the internalization of Lf-gold particles into the bacilli of *M.tb* H37Rv cells. Scale bar 0.1, 0.2 μM.

Both strains demonstrated cell surface binding of human Lf at 4°C that was competitively inhibited by the presence of 200X unlabeled Lf (Figures 3A,B). Lf binding was comparable in wild type and siderophore knockout strains (Figure 3C). Internalization of labeled Lf was also evident in both strains after incubation at 37°C (Figure 3D) indicating that GAPDH mediated Lf uptake is siderophore independent.

### Internalization of Lf into *M.tb* H37Rv

Bacilli were analyzed by TEM to assess the internalization of Lf-gold conjugated particles, which were observed within the cell cytosol (Figures 3E–G).

### Trafficking of Lf to intraphagosomal *M.tb*

**Figure 4:**
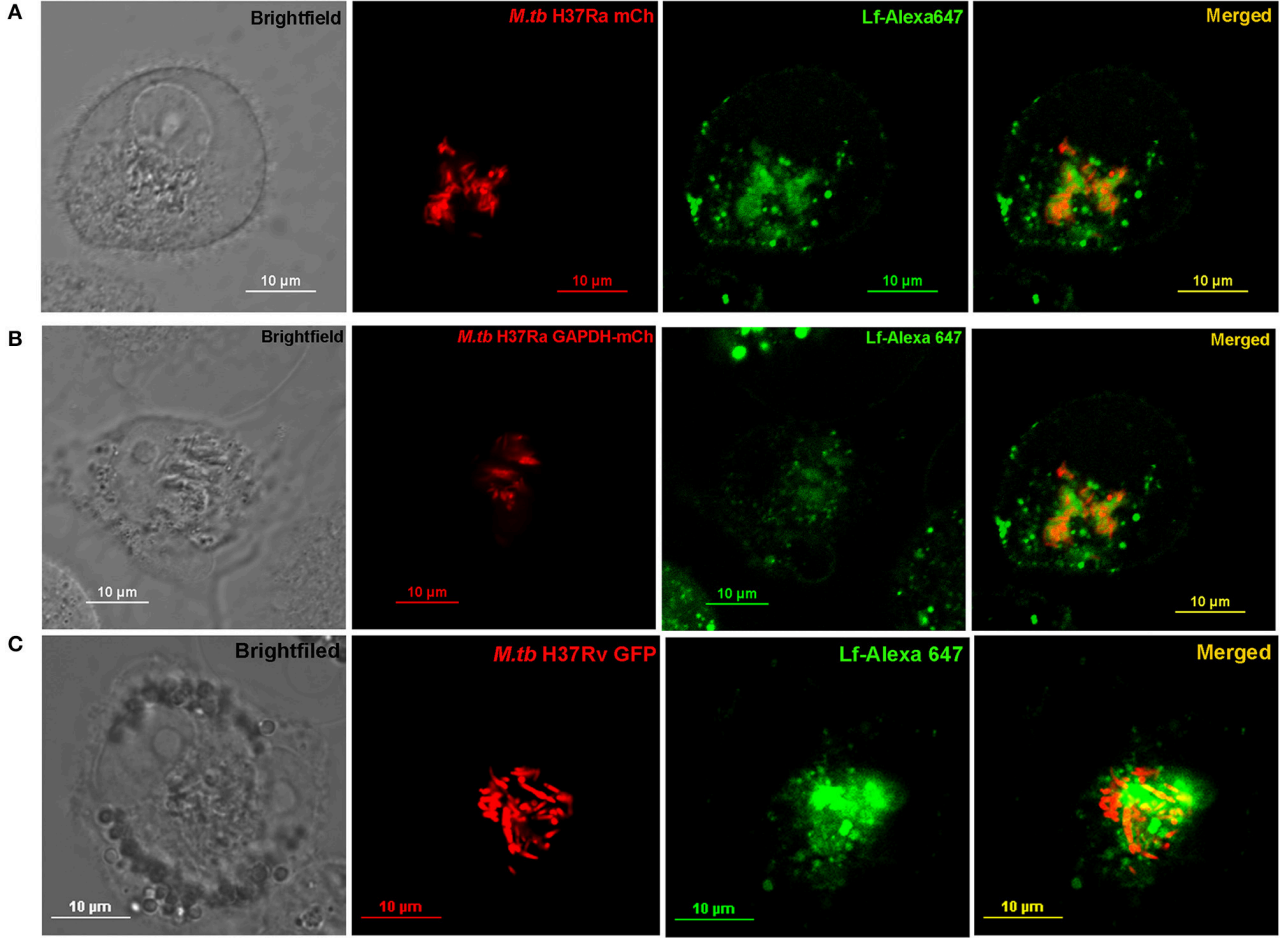
Trafficking of Lf to Intraphagosomal bacilli: THP-1 cells were infected with **(A)** *M.tb* H37Ra mCh **(B)** *M.tb* H37Ra GAPDH-mCh or **(C)** *M.tb* H37Rv GFP strains. The trafficking of Lf-A647 to the phagosome and co-localization with the intracellular bacteria are evident.

THP-1 cells were infected with either (i) *M.tb* H37Ra mCh (ii) *M.tb* H37Ra GAPDH-mCh or (iii) *M.tb* H37Rv-GFP strains (Figures 4A–C respectively). The trafficking of Lf-A647 to the intraphagosomal bacilli was assessed by confocal microscopy. In all cases the co-localization of Lf-A647 and bacilli was observed, live cell imaging of infected cells confirmed the endocytosis and subsequent co-localization of Lf with intraphagosomal *M.tb* H37Ra-GFP (Movie 1).

### Expression and purification of rGAPDH (N142S) and rGAPDH (P295L)

**Figure 5:**
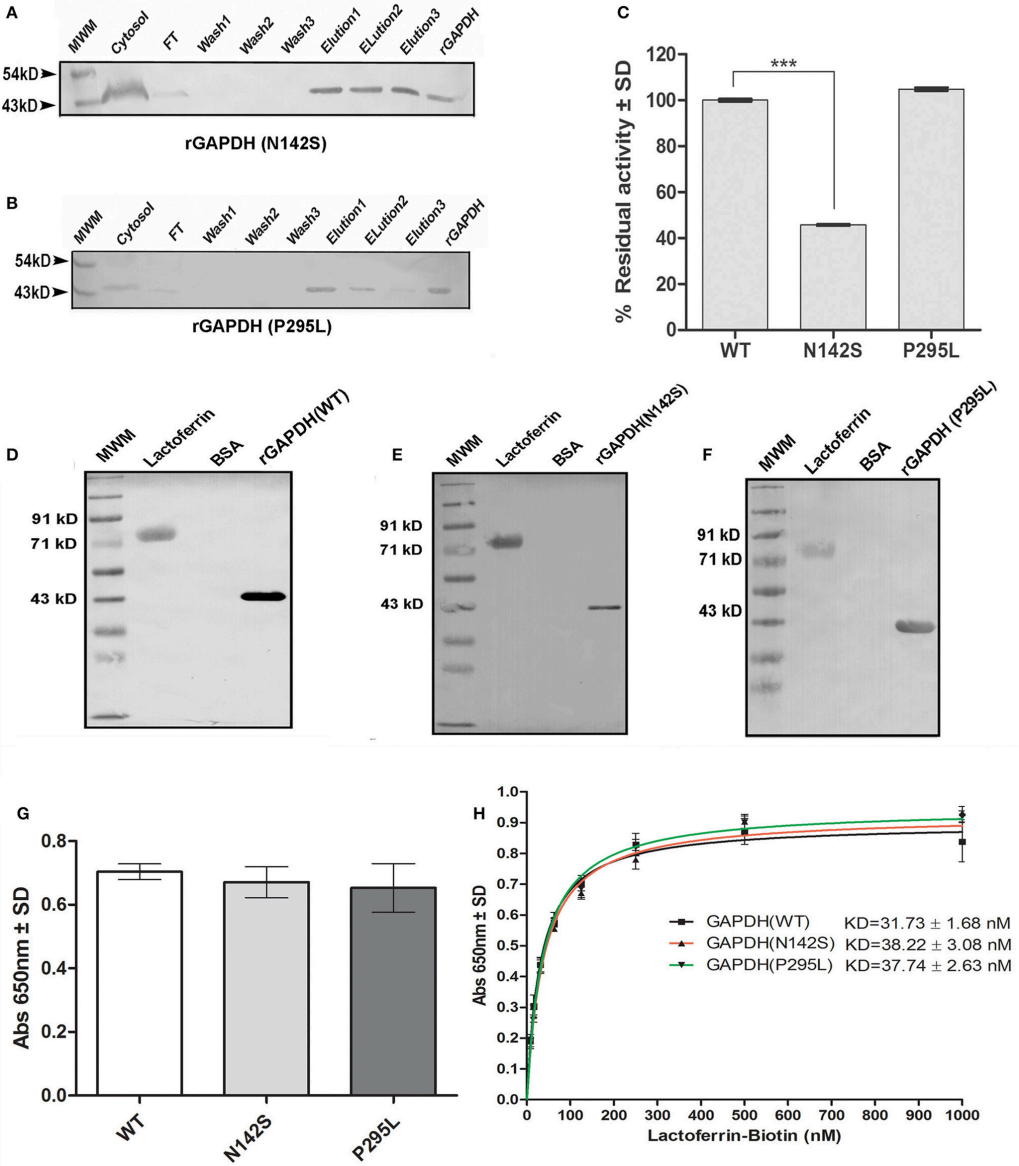
Analysis of *M.tb* GAPDH point mutations: Purification of recombinant GAPDH mutant proteins as confirmed by western blot **(A)** rGAPDH (N142S) **(B)** rGAPDH (P295L) **(C)** Enzyme activity of mutants and wild type protein. (*p* < 0.0001, *n* = 3) **(D–F)** Far western blotting assessed the interaction of Lf with rGAPDH (wt), rGAPDH (N142S), rGAPDH (P295L) respectively. **(G)** ELISA to estimate the binding of 125 nM Lf to wt rGAPDH, rGAPDH (N142S), rGAPDH (P295L) **(H)** Determination of affinity of interaction *(K*_*D*_*)* between rGAPDH, rGAPDH (N142S),or rGAPDH (P295L) with Lf.

Both proteins were expressed using *M.tb* H37Ra as a host strain. The proteins were purified using Ni-NTA affinity chromatography and purity confirmed by western blotting with mouse anti-His antibody (Figures 5A,B).

### Enzyme activity of mutants and interaction with Lf

The N142S mutant showed a significant loss of activity (*p* < 0.0001), only 45% enzyme activity was retained as compared to the wild type enzyme. In contrast the P295L mutant protein demonstrated enzyme activity comparable to wt rGAPDH (Figure 5C). The GAPDH-Lf interaction was confirmed *in vitro* by far western blotting using recombinant *M.tb* rGAPDH (rGAPDH) (Figure 5D). Resolved Lf was able to capture rGAPDH as evident by far western blotting, rGAPDH, and BSA were utilized as positive and negative controls respectively. The N142S, P295L mutant proteins also retained their ability to bind Lf in far western blots (Figures 5E,F). ELISA was carried out using 125 nM Lf to obtain quantitative data, no significant difference was observed between the three proteins (Figure 5G).

### *M.tb* GAPDH and Lf binding affinity

Lf binding to rGAPDH and mutants was observed to steadily increase with increasing concentration of Lf with saturation at 500 nM (Figure 5H). The *K*_*D*_ value of GAPDH-Lf interaction was estimated to be 31.7 ± 1.68 nM, a 5-fold higher affinity as compared to *M.tb* GAPDH and Tf (160 ± 24 nM). No significant difference in the affinity values were evident between wt and mutant proteins values. The N142S and P295L mutants were 38.22 ± 3.08 and 37.74 ± 2.63 nM respectively (Figure 5H).

### Sequence conservation of GAPDH

**Figure 6:**
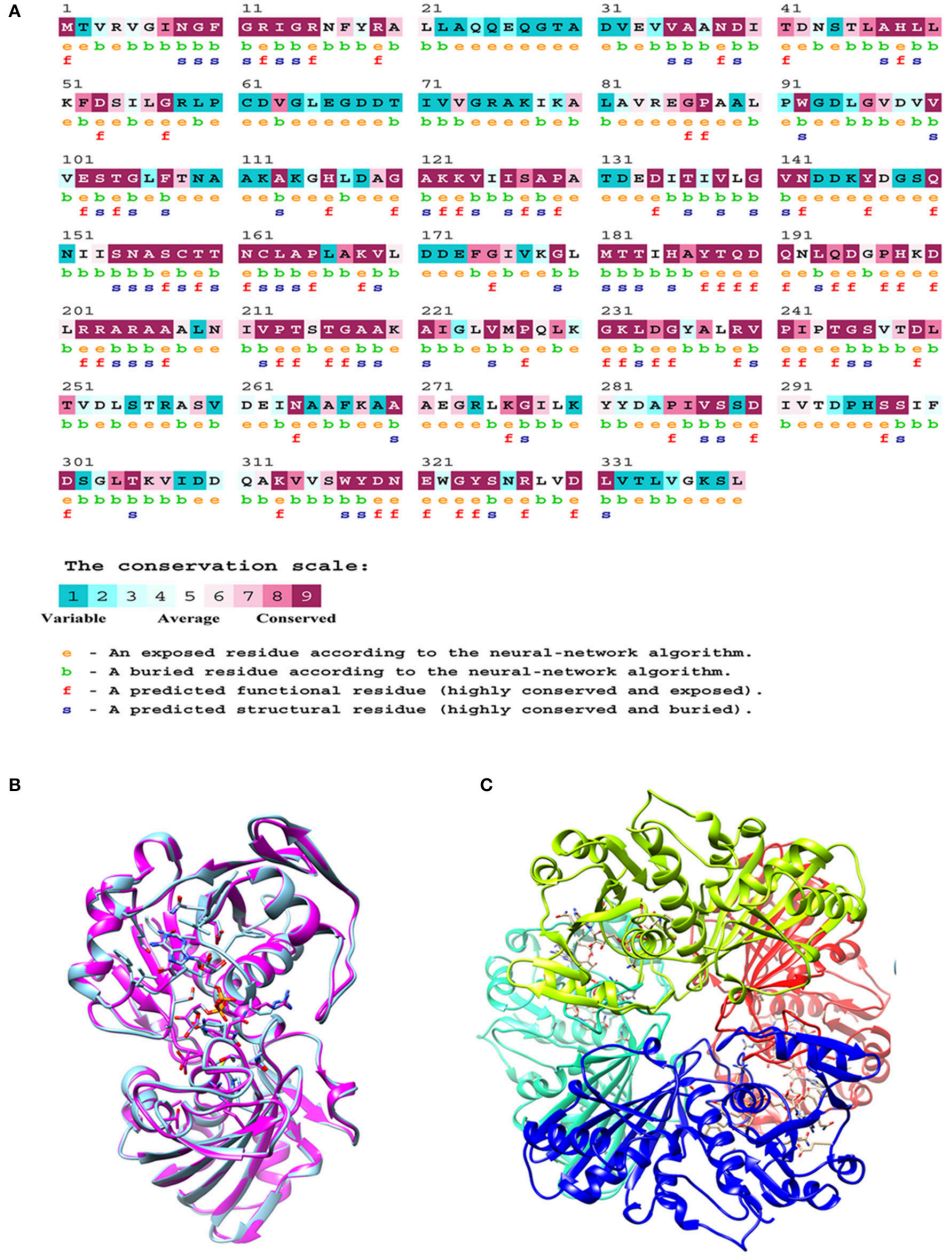
*In silico* analysis of *M.tb* GAPDH: **(A)** Consurf analysis to determine functionally and structurally important residues. **(B)** Modeled structure of GAPDH monomer. **(C)** Homology model of tetrameric *M.tb* H37Rv GAPDH based on GAPDH template from *Bacillus stearothermophilus* (PDB ID: 1GD1).

The *M.tb* GAPDH sequence was analyzed for its evolutionary conservation, it was observed that key active site residues as well as other surface exposed residues are highly conserved. Since it is known that GAPDH from multiple species binds to Tf (Modun and Williams, 1999), there is a high probability that the conserved regions (including active sites, co-factor binding site, and surface regions) may contribute to the interaction between Lf and Tf. Consurf server based evolutionary conservation analysis was carried out for the wt GAPDH sequence and the two point mutations N142S and P295L (http://consurf.tau.ac.il/2016/). Analysis of Asparagine 142 (N142) revealed that it is a highly conserved and exposed functional residue while Proline at 295 position is an exposed but variable residue (Figure 6A).

### Comparative homology modeling of GAPDH and mutant analysis

The comparative modeling of *M.tb* GAPDH was performed, using highly identical template of GAPDH from *B. stearothermophilus* (PDB ID: 1GD1). The two sequences demonstrate a sequence identity of 60%. The modeled 3D structure of GAPDH was validated by the RAMPAGE Ramachandran plot analysis server (http://mordred.bioc.cam.ac.uk/rapper/rampage.php). The quality of protein folds of GAPDH homology model was evaluated using ProSA protein structure analysis web server (https://prosa.services.came.sbg.ac.at/prosa.php). Modeled 3D structure aligned with the template (PDB ID: 1GD1) and assembled structural model was developed using UCSF Chimera software (https://www.cgl.ucsf.edu/chimera/), the RMSD score was 0.238 (Figures 6B,C).

**Figure 7:**
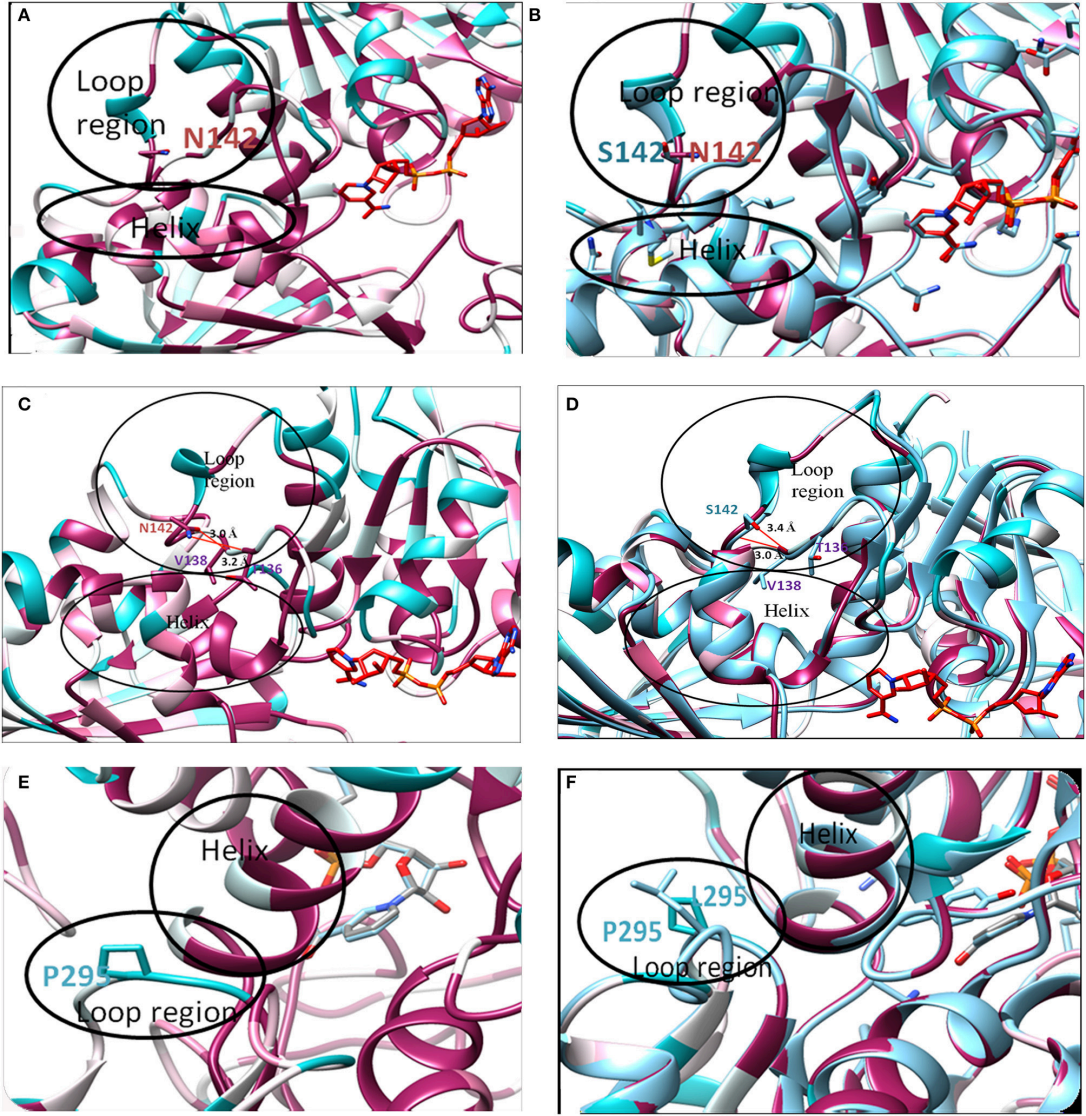
*In silico* analysis of mutations: Location of original and mutant residues **(A)** Model of Asparagine at position 142 of GAPDH **(B)** Structure showing the replacement of Asparagine with serine **(C)** Hydrogen bond formation is observed (represented as red line) between O-atom of Threonine 136 and NH_2_ group of Asparagine 142 (distance of interaction 3.2 Å). A second interaction occurs between the N-atom of Valine 138 and OH group of Asn 142 (distance of interaction 3.0 Å). **(D)** Formation of H-bond (represented as red line) is observed between O-atom of Valine 138 and N atom of Serine 142 (distance of interaction 3.0 Å) backbone interaction. A second interaction is observed between N-atom of Valine 138 and OH group of Serine 142 (distance of interaction 3.4 Å). As compared to the wild type Asparagine 142, the mutant Serine142 does not show any interaction with Threonine 136. **(E)** Model of Proline at position 295 of GAPDH **(F)** Structure showing the replacement of Proline with Leucine.

Although, *in silico* analysis of the mutant N142S suggests that this mutation may result in a non-significant structural change, since Serine at position 142 also shows a similar characteristic to the wild type residues N142, experimentally the mutation resulted in a loss of enzyme activity. Several computational methods have been developed for predicting the stability of mutant proteins based on sequence, structure, and Gibbs free energy estimation, this combined feature based approach provides better prediction of changes due to mutations (Kulshreshtha et al., 2016). In the current study, two different methods were used to predict the stability change of mutated proteins, predictions also assessed the sequence conservation. A combined sequence conservation and protein modeling study indicates Asparagine (N142) is a highly conserved and exposed residue that is located in a flexible loop region of GAPDH. Predicted stability change energies in the mutant (ΔΔG) indicate destabilization (Table 1). Structural analysis indicates that in *M.tb* GAPDH, the Glycine-Valine-Asparagine tripeptide sequence (at positions 140, 141, and 142 respectively) is also highly conserved in several GAPDH homologs. Additionally, it was observed that Asparagine 142 also has hydrogen bonding interactions with another highly conserved tripeptide region comprised of residues 136 to 138 (Figures 6, 7C). This tripeptide is composed of the sequence Threonine136-X-Valine138 where X indicates a variable residue. In *M.tb* GAPDH the intervening residue is Isoleucine at position 137 (Figure 6). Replacement of Asparagine with serine results in a loss of hydrogen bonding with Threonine 136 (Figure 7D). Computational modeling of the N142S mutant revealed conformational changes in this region during loop refinement, suggesting that this mutation may alter the stability of the molecule or its interaction with a helix that lies in close proximity (Figures 7A,B). While biochemical assays confirm a loss of enzyme activity, detailed analysis by co-crystallization, molecular modeling, extensive dynamic simulation, and mutagenesis would be essential to ascertain the relevance of these residues (if any) in the GAPDH-Lf/Tf interaction. In contrast the Proline 295 residue occurs in a variable loop region adjoining a highly conserved helix, mutation to Leucine does not result in a structurally significant change as confirmed by experimental data (Figures 7E,F).

**Table 1:** Analysis of mutants by computational methods.

**Mutations (predicted stability change ΔΔG)**	**mCSM**	**SDM**	**DUET**	**Conservation score**
N142S	−1.216	−0.36	−1.223	Highly (8–9)
P295L	−0.994	0.67	−0.793	Lower (1–2)

*ΔΔG in Kcal/mol, negative values indicate destabilization and positive values indicate stability of protein structure*.

*mCSM: Predicts the effect of mutations in proteins using graph-based signatures*.

*SDM: Site Directed Mutator is a statistical potential energy function method*.

*DUET: Protein Stability Change upon Mutation methods*.

## Discussion

Due to its intracellular location, *M. tuberculosis* faces a unique challenge in acquiring iron from within the host cell. It adopts multiple strategies to acquire this essential micronutrient from host iron carrier/storage proteins such as Tf, Lf, ferritin, and hemoglobin. Among these, two well-established processes involve the synthesis of siderophores and hemophores (De Voss et al., 2000; Tullius et al., 2011). Tf and Lf are conserved iron carrier proteins that share a high degree of sequence identity (60%), they are also structurally conserved. Iron acquisition from human Tf and Lf by the siderophores mycobactin and carboxymycobactin is well-characterized. Iron is chelated by the siderophore and transported via specific iron regulated transporters (Ryndak et al., 2010; Banerjee et al., 2011). However, the recombinant strains *M.tb* H37Rv Δmbt:: hyg and BCG(mbt)30 that do not express siderophores retain the ability to acquire Tf iron *in vivo*, indicating the presence of a siderophore independent pathway (De Voss et al., 2000; Tullius et al., 2008). Similar studies with Lf have not been evaluated. Although *M.tb* thrive in the lung, an environment rich in Lf rather than Tf, relatively less is known about Lf mediated iron uptake by *M.tb*. While previous studies have demonstrated that *M.tb* acquires iron from Lf in broth cultures and also within host macrophage cells the exact process is yet to be defined.

In pathogens such as *S.aureus, S.epidermidis, Neisseria gonorrhea, N.meningitidis, H. influenzae* iron acquisition from Tf and Lf can occur by the direct capture of these proteins by specific surface receptors (Williams and Griffiths, 1992; Gray-Owen and Schyvers, 1996; Modun and Williams, 1999; Taylor and Heinrichs, 2002). Recently our group identified multiple cell surface receptors for human Tf on the surface of *M.tb*. These include evolutionarily conserved metabolic enzymes such as GAPDH and LDH (Boradia et al., 2014). GAPDH is a highly conserved glycolytic enzyme that is present in all organisms. In human macrophages GAPDH functions as a dual receptor for both Tf and Lf (Raje et al., 2007; Rawat et al., 2012), moreover *M.tb* and human GAPDH are ~45% identical. Considering all these features we evaluated whether *M.tb* GAPDH can also function as a dual receptor for Lf. Preliminary studies using *M.tb* H37Ra cells revealed the specific binding of Lf to the bacterial surface. *In vitro* experiments confirmed that GAPDH is the receptor involved and overexpression of GAPDH in *M.tb* H37Ra cells resulted in a significant increase in binding and uptake of Lf as well as incorporation of associated iron. As reported by us in earlier studies involving GAPDH-Tf uptake (Boradia et al., 2014), Lf was internalized into the bacterium. At present the detailed mechanism of this process remains undefined.

Lf binding and uptake was also evident on the surface of virulent *M.tb* H37Rv and on the *M.tb* H37RvΔmbtB::hyg strain that is incapable of synthesizing siderophores. These findings are in agreement with previous reports demonstrating the intracellular survival and replication of siderophore negative strains within macrophages and in mice (De Voss et al., 2000; Tullius et al., 2008) indicative of the presence of alternate iron acquisition pathways.

To confirm whether this mechanism operates to deliver Lf to intraphagosomal bacilli, infected THP-1 cells were used as a model system. Confocal microscopy and live cell imaging studies clearly reveal the endocytosis and eventual co-localization of labeled Lf to the bacilli. Previous studies have evaluated the uptake of iron from endogenous and exogenous sources. In the endogenous model macrophages were preloaded with iron from Tf, Lf, or chelates and subsequently infected with *M.tb*. Iron uptake by the macrophages was comparable, but iron acquisition by *M.tb* was marginally more in cells preloaded with Lf-iron. In the case of experiments utilizing the exogenous model, cells were first infected with *M.tb* and then provided exogenous Tf, Lf, or Fe-citrate. When iron uptake by resident intracellular bacilli was evaluated, it was found that *M.tb* preferentially acquired Lf-iron as compared to Tf or Fe-citrate. Infected macrophages acquired 30-fold more iron from Lf and 3-fold more iron from citrate as compared to Tf. This suggests that if Lf and Tf are present in the external milieu, Lf is vastly more efficient at iron delivery to intra-phagosomal *M.tb* (Olakanmi et al., 2002, 2004). Our current study revealed that *M.tb* GAPDH possesses a 5-fold greater affinity for Lf as compared to Tf. Moreover, the Lf based iron uptake in the GAPDH mCh strain was evident within 1 h as opposed to 3 h in the case of Tf-iron (Boradia et al., 2014).

The crystal structure of *M.tb* GAPDH and structural details of the Lf/Tf-GAPDH interaction are at present unknown. In an effort to identify the key residues that may be involved in this interaction, analysis of two GAPDH mutants was carried out. Mutations of Asparagine at position 142 to Serine (N142S) and a Proline to Leucine alteration at position 295 (P295L) were assessed for their enzyme activity and ability to bind Lf. The N142S mutation demonstrated a drastic loss of enzyme activity as compared to wt GAPDH. The affinity of wild type protein and mutants for Lf were comparable (Figure 5H). This finding suggests that the two functions of GAPDH i.e., enzyme activity and Lf binding are independent of each other. Finally, the position of these residues within the GAPDH molecule was evaluated by *in silico* approaches. The sequence was evaluated for conserved, variable, exposed, or internal residues; a homology model was constructed for both the monomer and the tetrameric enzyme. It was found that N142 is a highly conserved, exposed residue that is present in a flexible loop region of GAPDH. Moreover, in *M.tb* GAPDH, the Glycine-Valine-Asparagine tripeptide sequence (at positions 140, 141, and 142 respectively) is highly conserved in several GAPDH homologs. Computational analysis suggested that this mutation may alter the stability of the molecule or its interaction with a helix that lies in close proximity (Figures 7A,B). Analysis also revealed that the interaction of N142 with conserved residues T136 and V138, hydrogen bonding with T136 was disrupted in the N142S mutant (Figures 7C,D), which may contribute to the observed loss of enzyme activity. However, detailed analysis by co-crystallization, molecular modeling, dynamic simulation, and mutagenesis would be necessary to ascertain the exact residues involved in the GAPDH-Lf/Tf interaction.

It is known that internalization of Tf into mammalian cells including macrophages can occur via the three identified receptors TfR1, TfR2 (absent on macrophages), and GAPDH (Jandl et al., 1959; Kawabata et al., 1999; Raje et al., 2007). Cell surface and soluble GAPDH are well-characterized routes for Lf uptake in macrophages (Rawat et al., 2012; Chauhan et al., 2015). Other known Lf receptors are Lipoprotein receptor-related protein (LRP) (Grey et al., 2004) and CD14 (Baveye et al., 2000). In the context of *M.tb* infection, further studies are essential to explore which of these pathways deliver iron to the phagosome and where the iron carrier molecules are transferred to the bacterial receptors. An alternate possibility is that unidentified, secreted bacterial proteins may intersect the endocytic pathway to capture and recruit Lf to the phagosome.

In conclusion, the current study reveals for the first time that *M.tb* can efficiently acquire iron from Lf utilizing GAPDH. These findings are relevant since lung fluids are rich in Lf and it is also an essential component of the innate immune response. The use of GAPDH as a single receptor that can acquire iron from both Tf and Lf indicates the ability of the bacilli to adapt to the external milieu. This study also brings into consideration the complex inter-relationship between the host and pathogen wherein GAPDH from both sources play parallel roles in iron acquisition. In siderophore mediated iron uptake, the host synthesizes siderocalins as a protective mechanism to inhibit iron uptake (Goetz et al., 2002; Flo et al., 2004; Berger et al., 2006). Utilizing its GAPDH for hijacking host iron provides an ideal astute strategy that offers a means of camouflage allowing the bacterium to operate this critical pathway undetected by the host cell. Unraveling the contribution of host and bacterial components in this alternate pathway could offer an insight into iron acquisition by *M.tb* and perhaps other pathogens.

## Availability of data and supporting material

The data supporting the research findings are available in the manuscript.

## Author contributions

CR and MR initiated the project. HM, AP, VB, MR, and CR designed the experiments, analyzed the data, and prepared the manuscript. RKu, RN, and PG designed and analyzed the *in silico* component of this study. HM, AP, VB, AK, and ZG were involved with reagent preparation, data acquisition, and analysis. RKa, contributed to reagent preparation and standardization.

### Conflict of interest statement

The authors declare that the research was conducted in the absence of any commercial or financial relationships that could be construed as a potential conflict of interest.

## Supplementary material

Movie 1 Trafficking and co-localization of Lf-A647 with intraphagosomal M.tb H37Ra-GFP.
